# Delayed endoscopic retrieval of a retained toothbrush: a case report of a rare 3-year asymptomatic retention

**DOI:** 10.1093/jscr/rjag002

**Published:** 2026-01-25

**Authors:** Hala Abdallah, Ameer Ameen, Rayan Yousif, Ahmed Rafei, Mohammed Ganim, Ghssan Abulgasim, Rawan A Bedab, Reem Salah, Abdelmoneim Eltayeb Abdo

**Affiliations:** Research Department, National Center for Gastrointestinal and Liver Diseases, 17 Street, Amarat, Khartoum 2, Khartoum State, PO Box 12217, 15004, Sudan; Research Department, National Center for Gastrointestinal and Liver Diseases, 17 Street, Amarat, Khartoum 2, Khartoum State, PO Box 12217, 15004, Sudan; Research Department, National Center for Gastrointestinal and Liver Diseases, 17 Street, Amarat, Khartoum 2, Khartoum State, PO Box 12217, 15004, Sudan; Research Department, National Center for Gastrointestinal and Liver Diseases, 17 Street, Amarat, Khartoum 2, Khartoum State, PO Box 12217, 15004, Sudan; Research Department, National Center for Gastrointestinal and Liver Diseases, 17 Street, Amarat, Khartoum 2, Khartoum State, PO Box 12217, 15004, Sudan; Research Department, National Center for Gastrointestinal and Liver Diseases, 17 Street, Amarat, Khartoum 2, Khartoum State, PO Box 12217, 15004, Sudan; Research Department, National Center for Gastrointestinal and Liver Diseases, 17 Street, Amarat, Khartoum 2, Khartoum State, PO Box 12217, 15004, Sudan; Research Department, National Center for Gastrointestinal and Liver Diseases, 17 Street, Amarat, Khartoum 2, Khartoum State, PO Box 12217, 15004, Sudan; Department of Gastroenterology, Ibn Sina Hospital, 17 Street, Amarat, Khartoum 2, Khartoum State, PO Box 12217, 15004, Sudan

**Keywords:** toothbrush ingestion, foreign body, endoscopy, gastrointestinal tract, conscious sedation, case report

## Abstract

Foreign body ingestion is a common clinical issue, but swallowing a full-sized toothbrush is exceptionally rare. We report a case of a 32-year-old Sudanese male with a three-year history of an accidentally ingested toothbrush. The patient presented with mild epigastric pain and intermittent dysphagia but no acute complications. Imaging, including X-ray and computed tomography, failed to identify the foreign body. Esophagogastroduodenoscopy revealed a complete toothbrush lodged in the stomach, with its head extending into the duodenum. The object was successfully removed intact using a polypectomy snare under conscious sedation, without mucosal injury or complications. This case highlights the diagnostic challenges posed by radiolucent foreign bodies and underscores the key role of endoscopy in both diagnosis and treatment. It also demonstrates that delayed endoscopic removal can be safely accomplished, though early retrieval remains essential to prevent serious outcomes such as perforation or obstruction.

## Introduction

Foreign body ingestion is a common clinical problem, most frequently encountered in children and in adults with psychiatric disorders, eating disorders, or suicidal intent [1].

Toothbrush ingestion is exceedingly rare and usually involves broken fragments rather than an intact brush [2]. Because of its length (>10 cm) and rigidity, a toothbrush is highly unlikely to traverse the fixed C-shaped duodenal loop [3]. Although many ingested objects pass spontaneously, elongated or rigid items may cause significant complications such as obstruction, perforation, or pressure necrosis [3].

Only a few dozen cases of toothbrush ingestion have been reported globally, most of which required endoscopic or surgical intervention [4].

We describe a rare case of a 20 cm full-sized adult toothbrush retained for approximately three years in an adult without psychiatric illness, successfully removed endoscopically.

## Case presentation

A 32-year-old Sudanese male, with no significant medical or psychiatric history, presented to the Gastroenterology Department with a 2-week history of dull, non-radiating epigastric pain unrelated to meals. He also reported intermittent choking episodes and mild dysphagia during the preceding two days. On examination, he was afebrile, hemodynamically stable, and abdominal examination revealed mild epigastric tenderness without guarding, rigidity or any sign of obstruction.

The patient recalled that 3 years earlier, he accidentally swallowed a manual 20 cm adult full-sized toothbrush while brushing his tongue with his head tilted backward. Initially asymptomatic, he did not seek medical care for 3 months. A healthcare consultation at that time included advice for imaging, but he left the facility before evaluation was completed.

**Figure 1:**
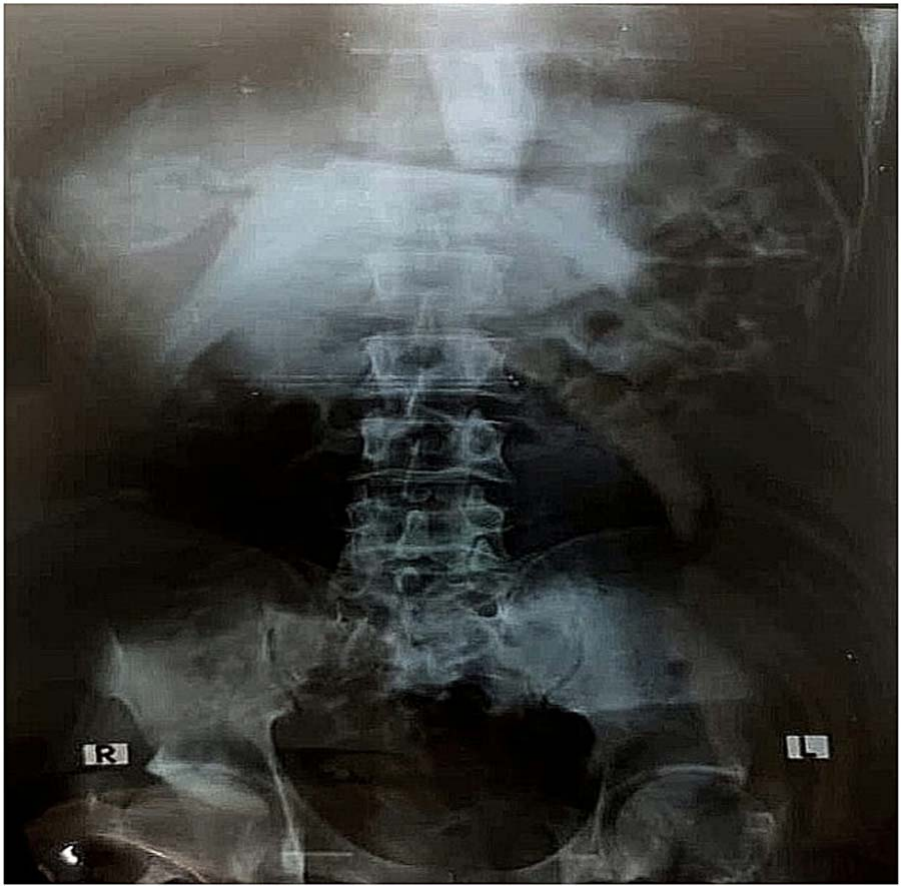
Abdominal X-ray showing no visible foreign body.

**Figure 2:**
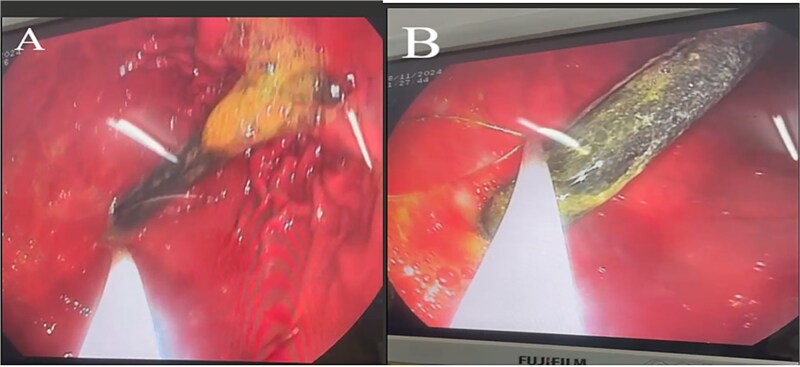
EGD demonstrating a manual toothbrush lodged in the stomach, with its head extending into the first part of the duodenum (D1). (A) The toothbrush head embedded within the gastric folds, partially coated with bile and food residue. (B) The elongated handle visible along the gastric body.

On current presentation, an abdominal X-ray (Fig. 1) showed no visible foreign body. Esophagogastroduodenoscopy (EGD) at a peripheral center (Fig. 2A and B) revealed a toothbrush lodged in the stomach with its head extending into the first part of the duodenum (D1); retrieval failed due to limited equipment.

**Figure 3:**
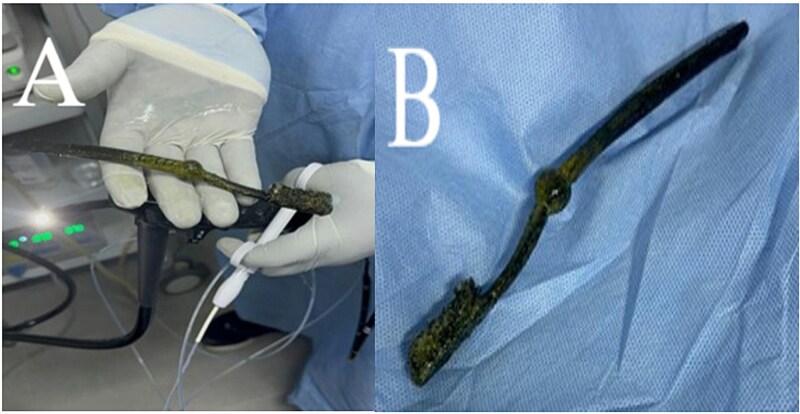
Endoscopic retrieval of the toothbrush using a polypectomy snare. (A) The snare securely grasping the toothbrush after removal. (B) The extracted toothbrush, removed in one piece without mucosal injury.

He was referred to our tertiary center for definitive management. Contrast-enhanced abdominal computed tomography (CT) showed no radiopaque foreign body or complications such as obstruction or perforation. A follow-up diagnostic and therapeutic gastroscopy under conscious sedation (intravenous midazolam 5 mg) identified the complete toothbrush with its handle in the stomach and head in D1. Using a 25 mm polypectomy snare, the toothbrush was extracted intact without mucosal injury (Fig. 3A and B). The procedure lasted 15 minutes and was well tolerated.

The patient was observed for 2 hours, and discharged the same day in stable condition. No delayed complications were reported on follow-up.

## Discussion

Foreign body ingestion represents a frequent challenge in emergency and gastroenterology practice; however, ingestion of large objects such as toothbrushes is exceptionally rare. To date, only a limited number of such cases have been documented [4].

Toothbrushes are typically over 10 cm in length and rigid, rendering spontaneous passage beyond the duodenal curvature unlikely, the prolonged retention of 3 years exceeds most reported durations, which typically range from hours to a few months [4–6]. In our patient, the head of the toothbrush was lodged in D1 while the handle remained in the gastric lumen, allowing long-term retention without progression or perforation.

Reported complications from retained toothbrushes include mucosal erosion, gastric perforation, abscess formation, and gastrointestinal bleeding [6, 7]. Our case is notable for the prolonged asymptomatic period, emphasizing the variable presentation and potential for diagnostic delay.

Imaging findings can be inconsistent. While toothbrushes may appear as faint linear metallic densities on plain radiographs due to bristle staples [8], detection often fails on both X-ray and CT — as in our case — because of low radiodensity and artifact interference. This underscores the superiority of EGD for both diagnosis and removal.

Endoscopic extraction is the first-line management for ingested foreign bodies. The earliest successful endoscopic retrieval of a toothbrush was reported in 1983 [9]. While most reported extractions are performed under general anesthesia, this case demonstrates that conscious sedation can be adequate for cooperative patients when airway protection and retrieval expertise are ensured [10].

Despite the absence of complications in this case, delayed removal carries a substantial risk of perforation and peritonitis [3, 6]. Therefore, early endoscopic intervention remains imperative once ingestion is suspected or confirmed.

## Conclusion

This rare case of long-term asymptomatic toothbrush retention illustrates that delayed endoscopic retrieval can be safely achieved without complications. Clinicians should maintain vigilance for foreign body ingestion in patients with unexplained gastrointestinal symptoms, even when imaging is negative. Early diagnosis and prompt endoscopic intervention remain the cornerstone of management. If endoscopic extraction fails or perforation occurs, surgical retrieval via laparoscopic gastrotomy is the recommended alternative.
